# Editorial: Stem Cell-Based Therapy in Retinal Degeneration

**DOI:** 10.3389/fnins.2022.879659

**Published:** 2022-03-25

**Authors:** Xiaona Huang, Hui Gao, Haiwei Xu

**Affiliations:** ^1^Southwest Hospital/Southwest Eye Hospital, Third Military Medical University (Amy Medical University), Chongqing, China; ^2^Key Lab of Visual Damage and Regeneration and Restoration of Chongqing, Chongqing, China

**Keywords:** stem cell, retinal degeneration, transplantation, microenvironment, organoids

Retinal degeneration (RD), consisting of a group of heterogeneous diseases, causes the visual loss of millions of patients worldwide, the typical pathological changes of RD are degeneration and death of photoreceptors and retinal pigment epithelium (RPE) cells, which fail to regenerate (Sahel et al., 2014). Among current therapeutic strategies, stem cell-based therapy has become a new perspective for the treatment of RD, as stem cells are characterized with the strong abilities of self-renewal, multi-directional differentiation, neuroprotection, and immuno-regulation (West et al., 2020; Holan et al., 2021).

Based on this topic, the compilation includes five original research articles and one review article, exploring the therapeutic effects and mechanisms of stem cell-based therapy from diverse aspects and providing some new insights. According to the method and mechanism, it can be divided into three aspects: trophic support microenvironment, integration, and disease model establishment.

## Trophic Support Microenvironment

Stem cells can improve the retinal microenvironment by secreting trophic factors and regulating the immune response to promote vascular repair and cell survival (Jin et al., 2019; Yamanaka, 2020).

The senescence of RPE is the characteristic and pathogenic factor of age-related macular degeneration (AMD). Wang et al. showed that coculturing with embryonic stem cells (ESCs) contributed to the proliferation and the senescence-related markers downregulation of premature and replicative aging RPE cells, that is, rejuvenation, to reverse the aging of RPE. And the research has proved that ESCs reverse the senescence of RPE mainly through activating PI3K and TGFβ pathways. This result contributes to the use of stem cells for the AMD treatment by creating a younger environment.

Noueihed et al. are focusing on ischemic retinopathy (IR), a disease characterized by the microvascular destruction and pathological neovascularization formation (Bertelli et al., 2020). Mesenchymal stem cells (MSCs) were found to regulate the immune microenvironment of ischemic retina and promote the retinal revascularization in oxygen-induced retinopathy (OIR) model by restoring the neuronal Sema3E level and reducing the pathological level of IL-17A in myeloid cells. This article revealed the unprecedented interaction among MSCs, neurons, and myeloid cells, and established a healthy microenvironment that allowed ischemic retinal vascular regeneration.

Chen et al. present work on the vascular protection and neurotrophic effects of transplanted stem cells, which were used to protect retinal endothelial cells and retinal ganglion cells (RGCs) of X-ray irradiated rat retina. After CD133^+^CD34^+^ cells from human umbilical cord blood (hUCB-CD133^+^CD34^+^) cells were transplanted into the eyes of rats with radiation retinopathy, it was observed that the visual function, vascular function, and the survival rate of RGCs were improved significantly. It is proved that this is mainly due to the neurotrophic factors and anti-apoptosis factors secreted by CD133^+^ CD34^+^ cells for improving the microenvironment.

The review of Lin et al. on this Research Topic is a systematic and comprehensive summary of the treatment of MSCs for retinal degenerative diseases in recent years. MSCs and retinal microenvironment can interact through cell communication to promote the survival of retinal cells and regulate inflammation and immune response. However, it also faces the challenges of overcoming MSCs heterogeneity and optimizing delivery methods to reduce adverse reactions.

## Stem Cell Integration

The advanced stages of RD is characterized by the massive loss of retinal neurons, therefore, the direct use of stem cells to replace the injured neurons is also a research hotspot (Nazari et al., 2015). A number of studies have shown that stem cells transplanted into the damaged retina can differentiate into retinal cells, and even integrate into the retina to restore the visual function of RD (Gagliardi et al., 2019; Jin et al., 2021).

Thomas et al. generated the co-grafts called “total retina patch” consisting of human ESCs derived retina organoids and RPE cells using a bio-adhesive method and established a novel cell therapy paradigm for irreversible retinal eye injury. By transplanting co-grafts into the retina of immunodeficient rats, the long-term survival of the grafts was observed, it demonstrated that the co-grafts produced various cell types including photoreceptor cells that integrated into the host retina, which improved the visual function. This strategy showed a better therapeutic effect than previous RPE transplantation alone (Seiler et al., 2017; McLelland et al., 2018).

## Disease Model Establishment of Stem Cell-Derived Organoids

Stem cell-derived organoids have become an important model for the study of retinal diseases, as they may be able to reproduce the morphological and molecular characteristics of the retinal diseases during development *in vitro* (Bell et al., 2020; O'Hara-Wright and Gonzalez-Cordero, 2020). Zeng et al. found that in the early development stage of human retina, Particulate Matter (PM2.5) exposure may inhibit cellular proliferation, increase cellular apoptosis and trigger mis-location of neurons through MAPK and PI3K/Akt pathways, resulting in abnormal development of human retina in hESC-derived retinal organoids.

## Conclusion

These research articles on the topic show that it is very promising to treat retinal degenerative diseases based on stem cell-therapy, and provide many new perspectives and ideas. It is expected that more articles will emerge and open a new gorgeous chapter of stem cells in the treatment of retinal degenerative diseases.

## Author Contributions

XH and HG prepared the manuscript. HX revised the manuscript and provided financial support. All authors read and approved the final manuscript.

## Funding

This study was supported by funding from the National Natural Science Foundation of China (No. 31930068).

## Conflict of Interest

The authors declare that the research was conducted in the absence of any commercial or financial relationships that could be construed as a potential conflict of interest.

## Publisher's Note

All claims expressed in this article are solely those of the authors and do not necessarily represent those of their affiliated organizations, or those of the publisher, the editors and the reviewers. Any product that may be evaluated in this article, or claim that may be made by its manufacturer, is not guaranteed or endorsed by the publisher.

## References

[B1] Bell C. M. Zack D. J. Berlinicke C. A. (2020). Human organoids for the study of retinal development and disease. Annu. Rev. Vis. Sci. 6, 91–114. 10.1146/annurev-vision-121219-081855 32936736

[B2] Bertelli P. M. Pedrini E. Guduric-Fuchs J. Peixoto E. Pathak V. Stitt A. W.. (2020). Vascular regeneration for ischemic retinopathies: hope from cell therapies. Curr. Eye Res. 45, 372–384. 10.1080/02713683.2019.1681004 31609636

[B3] Gagliardi G. Ben M'Barek K. Goureau O. (2019). Photoreceptor cell replacement in macular degeneration and retinitis pigmentosa: a pluripotent stem cell-based approach. Prog. Retin. Eye Res. 71, 1–25. 10.1016/j.preteyeres.2019.03.001 30885665

[B4] Holan V. Palacka K. Hermankova B. (2021). Mesenchymal stem cell-based therapy for retinal degenerative diseases: experimental models and clinical trials. Cells 10:588. 10.3390/cells10030588 33799995 PMC8001847

[B5] Jin N. Sha W. Gao L. (2021). Shaping the microglia in retinal degenerative diseases using stem cell therapy: practice and prospects. Front. Cell Dev. Biol. 9:741368. 10.3389/fcell.2021.741368 34966736 PMC8710684

[B6] Jin Z. B. Gao M. L. Deng W. L. Wu K. C. Sugita S. Mandai M.. (2019). Stemming retinal regeneration with pluripotent stem cells. Prog. Retin. Eye Res. 69, 38–56. 10.1016/j.preteyeres.2018.11.003 30419340

[B7] McLelland B. T. Lin B. Mathur A. Aramant R. B. Thomas B. B. Nistor G.. (2018). Transplanted hESC-derived retina organoid sheets differentiate, integrate, and improve visual function in retinal degenerate rats. Invest. Ophthalmol. Vis. Sci. 59, 2586–2603. 10.1167/iovs.17-23646 29847666 PMC5968836

[B8] Nazari H. Zhang L. Zhu D. Chader G. J. Falabella P. Stefanini F.. (2015). Stem cell based therapies for age-related macular degeneration: the promises and the challenges. Prog. Retin. Eye Res. 48, 1–39. 10.1016/j.preteyeres.2015.06.004 26113213

[B9] O'Hara-Wright M. Gonzalez-Cordero A. (2020). Retinal organoids: a window into human retinal development. Development 147:dev189746. 10.1242/dev.189746 33361444 PMC7774906

[B10] Sahel J. A. Marazova K. Audo I. (2014). Clinical characteristics and current therapies for inherited retinal degenerations. Cold Spring Harb. Perspect. Med. 5:a017111. 10.1101/cshperspect.a017111 25324231 PMC4315917

[B11] Seiler M. J. Lin R. E. McLelland B. T. Mathur A. Lin B. Sigman J.. (2017). Vision recovery and connectivity by fetal retinal sheet transplantation in an immunodeficient retinal degenerate rat model. Invest. Ophthalmol. Vis. Sci. 58, 614–630. 10.1167/iovs.15-19028 28129425 PMC6020716

[B12] West E. L. Ribeiro J. Ali R. R. (2020). Development of stem cell therapies for retinal degeneration. Cold Spring Harb. Perspect. Biol. 12, 1–23. 10.1101/cshperspect.a035683 31818854 PMC7397828

[B13] Yamanaka S. (2020). Pluripotent stem cell-based cell therapy-promise and challenges. Cell Stem Cell 27, 523–531. 10.1016/j.stem.2020.09.014 33007237

